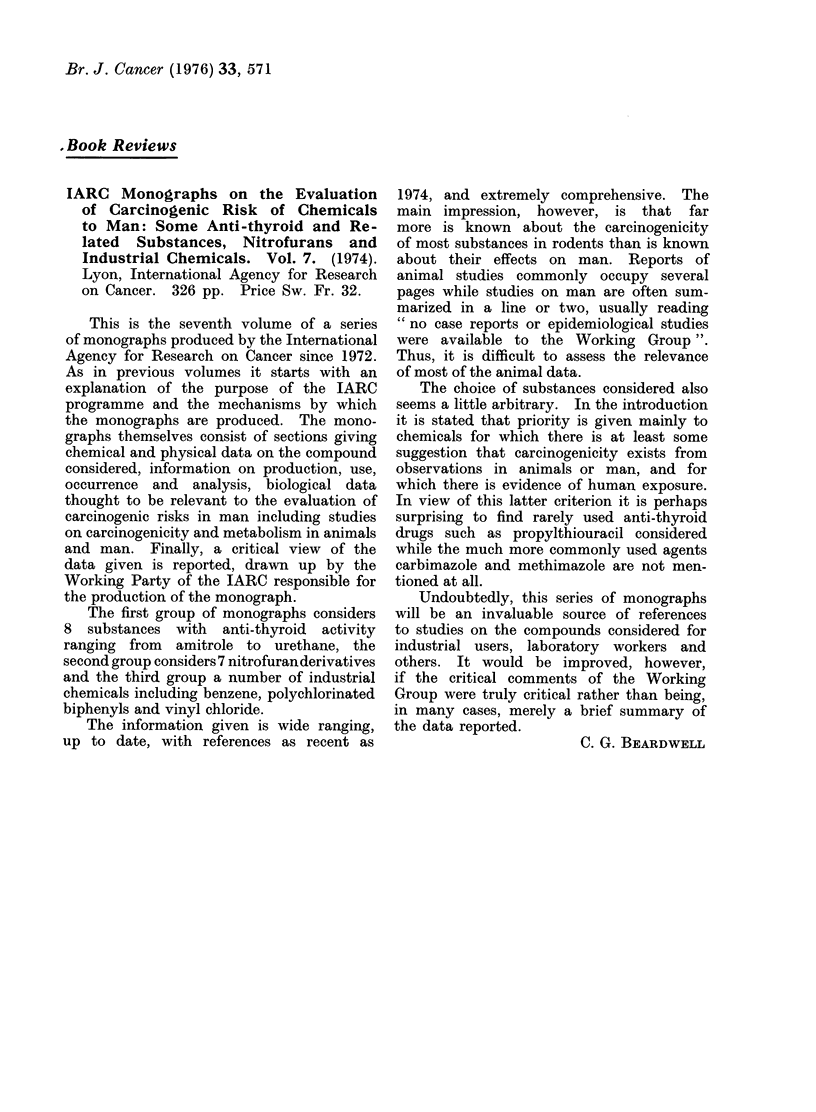# IARC Monographs on the Evaluation of Carcinogenic Risk of Chemicals to Man: Some Anti-thyroid and Related Substances, Nitrofurans and Industrial Chemicals

**Published:** 1976-05

**Authors:** C. G. Beardwell


*Br. J. Cancer* (1976) **33**, 571

### *Book Reviews*

**IARC Monographs on the Evaluation of Carcinogenic Risk of Chemicals to Man: Some Anti-thyroid and Related Substances, Nitrofurans and Industrial Chemicals. Vol. 7.** (1974). Lyon, International Agency for Research on Cancer. 326 pp. Price Sw. Fr. 32.

This is the seventh volume of a series of monographs produced by the International Agency for Research on Cancer since 1972. As in previous volumes it starts with an explanation of the purpose of the IARC programme and the mechanisms by which the monographs are produced. The monographs themselves consist of sections giving chemical and physical data on the compound considered, information on production, use, occurrence and analysis, biological data thought to be relevant to the evaluation of carcinogenic risks in man including studies on carcinogenicity and metabolism in animals and man. Finally, a critical view of the data given is reported, drawn up by the Working Party of the IARC responsible for the production of the monograph.

The first group of monographs considers 8 substances with anti-thyroid activity ranging from amitrole to urethane, the second group considers 7 nitrofuran derivatives and the third group a number of industrial chemicals including benzene, polychlorinated biphenyls and vinyl chloride.

The information given is wide ranging, up to date, with references as recent as 1974, and extremely comprehensive. The main impression, however, is that far more is known about the carcinogenicity of most substances in rodents than is known about their effects on man. Reports of animal studies commonly occupy several pages while studies on man are often summarized in a line or two, usually reading " no case reports or epidemiological studies were available to the Working Group ". Thus, it is difficult to assess the relevance of most of the animal data.

The choice of substances considered also seems a little arbitrary. In the introduction it is stated that priority is given mainly to chemicals for which there is at least some suggestion that carcinogenicity exists from observations in animals or man, and for which there is evidence of human exposure. In view of this latter criterion it is perhaps surprising to find rarely used anti-thyroid drugs such as propylthiouracil considered while the much more commonly used agents carbimazole and methimazole are not mentioned at all.

Undoubtedly, this series of monographs will be an invaluable source of references to studies on the compounds considered for industrial users, laboratory workers and others. It would be improved, however, if the critical comments of the Working Group were truly critical rather than being, in many cases, merely a brief summary of the data reported.

C. G. Beardwell